# Celecoxib Protects Hyperoxia-Induced Lung Injury via NF-κB and AQP1

**DOI:** 10.3389/fped.2019.00228

**Published:** 2019-06-07

**Authors:** Dongyun Liu, Yuguang Wang, Lili Li, Han Zhao, Liangliang Li, Yan Liu, Hong Jiang, Xianghong Li, Rui Zhang

**Affiliations:** ^1^Neonatal Intensive Care Unit, The Affiliated Hospital of QingDao University, Qingdao, China; ^2^Pediatric Department, Liaocheng City People's Hospital, Liaocheng, China; ^3^Department of Pathology, The Affiliated Hospital of QingDao University, Qingdao, China

**Keywords:** bronchopulmonary dysplasia, preterm infants, hyperoxia, celecoxib, NF-κB, AQP1

## Abstract

**Objective:** There is an increasing incidence of bronchopulmonary dysplasia (BDP) in preterm infants in China, which is the key issue affecting their survival rate and life quality. This study was performed to better understand the mechanism of protective effect of celecoxib on hyperoxia induced injury.

**Methods:** Hyperoxia BPD model was established using newborn Sprague-Dawley (SD) rats exposed to high O_2_ level (85%). Celecoxib treatment was also conducted. Histology of lung tissue samples were analyzed. Functional studies were systematically performed using the lung tissues and A549 cells.

**Results:** Hyperoxia disrupted lung development in SD rats. Celecoxib alleviated the damaged lung development. NF-κB and Aquaporin (AQP) 1 were identified as the pathways in the hyperoxia-induced lung injury. We have shown that hyperoxia activated NF-κB pathway through increased nucleus translocation and repressed AQP1 expression. On the contrary, celecoxib inhibited NF-κB phosphorylation and nucleus translocation and increased AQP1 expression through inhibiting COX2 activity. Additionally, celecoxib also rescued apoptosis induced by hyperoxia.

**Conclusion:** Our study identified NF-κB and AQP1 as the pathways in the hyperoxia-induced lung injury in the hyperoxia BPD model SD rats and it provided a better understanding of the protective effect of celecoxib. It suggests NF-κB and AQP1 may be as potential targets for treating newborns with BPD.

## Introduction

In recent years, the survival rate of preterm infants has been increasing with the advance of medical technology. However, there has been a rise of the incidence of bronchopulmonary dysplasia (BDP) in preterm infants who always require respiratory support, which became the key issue affecting their survival rate and life quality (1). Hyperoxia exposure in premature infants results in apoptosis, arrested septation and inflammation, which eventually leads to the development of BPD (2, 3). Currently, the treatment of BPD includes prenatal steroids, non-steroidal medicine and stem cell therapy (4). However, there has been little research on the Non-Steroidal Anti-inflammatory Drug (NSAID) as a preventative and reparative treatment for BPD.

COX2 is an inducible form of myeloperoxidases and plays an important role in inflammation and cancer. COX2 expression is induced by hyperoxia in mouse BPD models, which makes it a potential target for treating patients with BPD (5, 6). Celecoxib was developed as a NSAID which selectively inhibits COX2 activity (7). Compared with non-selective NSAIDS, celecoxib is well-tolerated with lower gastrointestinal toxicity. Complaints about mild or moderate headache, dyspepsia, upper respiratory tract infection, diarrhea, sinusitis, abdominal pain, and nausea were reported in patients with rheumatoid arthritis or osteoarthritis receiving celecoxib 200 or 400 mg/day. Since the withdrawn of rofecoxib and valdecoxib due to their increased cardiovascular risk. Attention was paid to the cardiovascular safety of celecoxib. Although one study reported a dosage related increase in cardiovascular risk with celecoxib 400 and 800 mg/day, no significant difference in cardiovascular risk of celecoxib comparing with placebo or non-selective NSAIDS was reported in any other trials. For patients who are at high risk for NSAID-induced GI toxicity, or have shown intolerance of NSAIDS, celecoxib is proven to be a more advantageous therapeutic option (7). Thus, celecoxib has been widely used to treat osteoarthritis, rheumatoid arthritis, acute pain, menstrual pain, ankylosing spondylitis, and to reduce the number of colorectal polyps in people with familial adenomatous polyposis, but it is not reported to treat infants with BPD (8, 9).

NF-κB regulates many physiological processes, such as inflammation, cell proliferation, apoptosis (10, 11). Oxidative stress activates NF-κB pathway, and activated NF-κB regulates the expression and activation of inflammatory factors and eventually leads to apoptosis, which is one of the characteristics of BPD (12–14). Aquaporin (AQP) 1 plays a major role in lung osmotic water permeability and have anti-pulmonary edema effect (15, 16). Reduction of AQP1 expression was noted in pulmonary injury (17). Moreover, previous study has been shown that AQP1 has a protective effect on cells against apoptosis (18).

Although previous studies have suggested COX2, NF-κB, and AQP1 are implicated in the development of lung, the cross-talk and interaction between these factors have not been fully revealed in animal model of BPD. In this paper, we demonstrated that celecoxib alleviated the lung injury and rescued alveolar apoptosis in detail through inhibiting COX2, suppressing NF-κB activation and increasing AQP1 expression, which presents a novel signaling pathway as therapeutic target.

## Materials and Methods

### Animal Model

This study was carried out in accordance with the recommendations of “the Guide for the Care and Use of Laboratory Animals,” as adopted and promulgated by the United States National Institutes of Health. The protocol was approved by the Animal Ethics Committee of the Affiliated Hospital of QingDao University [Approved by SCXK (Lu) 20130001]. SD rats (Qingdao Experimental Animal Center, China) were purchased and housed in a standard animal room.

One hundred five 2-day-old rats were randomly divided into three groups. Group I, control group: i.p. vehicle (DSMO) and normoxia (21% O_2_); Group II, hyperoxia group (BPD group): i.p. vehicle (DSMO) and hyperoxia (85% O_2_); Group III, hyperoxia and celecoxib group (BPD treatment group): i.p. celecoxib and hyperoxia (85% O_2_). Group I rats were continuously exposed to room air and fed by pregnant dams; Group II and III rats were separated from mother, fed with milk and placed in a self-made oxygen box with continuous input of oxygen which is at 850 ml/L and monitored by an oxygen sensor (LongFian Scitech, China). The food, water, and bedding were changed every morning. Intraperitoneal injection of celecoxib was performed daily at 5 mg/kg on group III. Group I and II rats were injected with same amount of DMSO as control. Experimental rats in three groups were randomly selected and anesthetized i.p. with ketamine and xylazine on day 3 (Group I: *N* = 10; Group II: *N* = 10; Group III: *N* = 10), day 7 (Group I: *N* = 10; Group II: *N* = 10; Group III: *N* = 10), and day 14 (Group I: *N* = 15; Group II: *N* = 13; Group III: *N* = 14) after being treated with or without hyperoxia and celecoxib (Selleck chemicals, US).

### Histologic Analyses, Morphometric Analysis, and Immunohistochemistry (IHC)

For histologic analyses, rats were euthanized. Lungs were inflated with 50% optimum cutting temperature (OCT) compound/50% PBS mixture via the trachea at 25 cm H_2_O and collected with care. The lung tissues were frozen in a disposable mold containing OCT with dry ice and isopentane slurry, then stored in −80°C freezer. Frozen sections were cut at 5 μm with a cryostat, mounted on Superfrost Plus microscope slides (Thermo Fisher Scientific, US). After fixation, the lung tissue slides were washed and stained with hematoxylin and eosin (H&E) (Beyotime Biotechnology, China). All slides were evaluated by a pathologist who was blind to the experimental.

Radial alveolar count (RAC) and the mean septal wall thickness (ST) were used to determine the effect of hyperoxia and celecoxib on lung development. Using image analysis, a perpendicular line was drawn between the respiratory bronchiole to the nearest connective tissue septum or lung pleural surface. RAC was measured for every bronchiole on a slide, and an average radial alveolar count was calculated. For ST measurement, images were imported into Microsoft powerpoint at 200 × magnification of the original images, and examined under a grid of five equally spaced horizontal lines. The ST was measured perpendicularly at the point where the alveolus crossed the horizontal line.

For immunohistochemistry, sections were blocked and incubated with anti- AQP1, anti-NF-κB (p65), anti-p-NF-κB (p65) antibodies (Abways Technology, China) overnight at 4°C. The next morning, sections were washed and incubated with Goat Anti-Rabbit IgG (Abways Technology, China) at room temperature for 1 h followed by washing three times. Quantification was performed using ImageJ (National Institutes of Health, US).

### Cell Line Culture Conditions and Cell Treatment

Human lung epithelial A549 cells were used as cell model (19, 20), A549 cells were purchased from Institute of Basic Medical Sciences Chinese Academy of Medical Sciences, and maintained in 1640 medium (Gibco-BRL, USA) supplemented with 10% fetal bovine serum (Gibco-BRL, USA) at 37°C and 5% CO_2_. Hyperoxia exposure of A549 cells was performed in a humidified chamber with continuous input of 85% oxygen and 15% of CO_2_ at 37°C.

### Immunoblotting

Lung tissues or A549 cells were homogenized in cold RIPA buffer supplemented with protease inhibitors, phosphatase inhibitors, sodium orthovanadate, and PMSF (Sigma-Aldrich, US). The nucleus and cytosol fractionations were performed using Nuclear Cytosol Fractionation Kit (Biopioneer Tech, China). The lysates were spun at 14,000 rpm for 10 min at 4°C, and protein was fractioned by SDS-PAGE. The gel was transferred to a PVDF membrane and incubated with anit-AQP1, anti-NF-κB (p65), anti-p-NF-κB (p65), anti-p-AKT (473), anti-AKT, anti-COX2 (Santa Cruz, US), and anti-caspase 3 (Millipore Sigma) antibodies overnight at 4°C. Membranes were then washed with T-BST and incubated with specific secondary antibodies for 1 h at room temperature and signal was detected using Supersignal West (Pierce, US).

### RNA Extraction and Quantitative Real-Time Polymerase Chain Reaction (PCR)

RNA of the lung tissues was extracted using TRIzol (Invitrogen, US) according to the manufacture's instruction. Deoxyribonuclease I (Roche Applied Science, US) was used to treat genomic DNA. One microgram RNA was used to synthesize the cDNA using GoScript™ Reverse Transcriptase (Promega, US) and real-time PCR were performed using the SsoFast™ EvaGreen Supermix®kit (Bio-Rad, US) according to manufacturer's protocol. *AQP1* primers: 5′tccctgctcgagaactcact3′ and 5′agagccacagacaagccaat3′, *GAPDH* primers: 5′CAACTCCCTCAAGATTGTCAGCAA3′ and 5′GGCATGGACTGTGGTCATGA3′. The relative expression was analyzed according to the 2-ΔΔCq method (21).

### Measurement of COX2 Activity

Lung tissue was washed and homogenized in cold tris buffer. Samples were spun down at 10,000 g for 15 min at 4°C, and supernatant were used for assay using the COX2 activity kit (Cayman Chemical, US) according to the manufacturer's protocol. In the end, the numbers were read using Molecular Devices Lmax luminometer microplate reader (Sunnyvale, US).

### Measurement of TNFα, Interleukin (IL)-6 Concentration in Bronchoalveolar Lavage Fluid (BALF)

Concentrations of TNFα, interleukin (IL)-6 in the supernatants of BALF were measured using enzyme-linked immunosorbent assay (ELISA) kits (R&D Systems, US) according to the manufacturer's protocol. Numbers were read using the microplate reader.

### PGE2 Release Measurement

A549 cells were cultured either in regular condition or exposed to hyperoxia for 72 h. In inhibitor pretreatment experiments, cells were treated with or without 10 μM of PDTC and 25 μM LY294002 for 1 h before hyperoxia exposure. The supernatant was harvested and diluted 10-fold with the enzyme immunoassay (EIA) buffer, and immediately assayed using the PEG2 ELISA assay kit (Cayman chemical, US) according to the manufacture's protocol and read using the microplate reader.

### Apoptosis Assay

Lung tissue slides were prepared as described earlier. The slides were pretreated, stained using SignalStain® Apoptosis (Cleaved Caspase-3) IHC Detection Kit (Cell Signaling, US) and washed according to the manufacturer's protocol. The slides were finally observed under an optical microscope. Fifteen sections were selected from each group. Five high magnification fields (×400) were randomly taken from each section and 100 cells were counted.

One hundred microliter of the A549 cells was added to an opaque 96 well-plate, 100 μl Celltiter-Glo (Promega, US) reagent mixture was added. The plate was placed on a shaker for 10 min. Numbers were read using the Celltiter-Glo protocol on microplate reader.

### Statistics

All data are provided as means ± SEM. Statistical differences between control group and hyperoxia group were analyzed by SPSS version 13.0 program (SPSS Inc., Chicago, IL, USA) using oneway analysis of variance (ANOVA). Statistical significance was defined by at *p* < 0.05.

## Results

### Celecoxib Improved Lung Development Disrupted by Hyperoxia in the Experimental Animal Model of BPD

**Figure 1:**
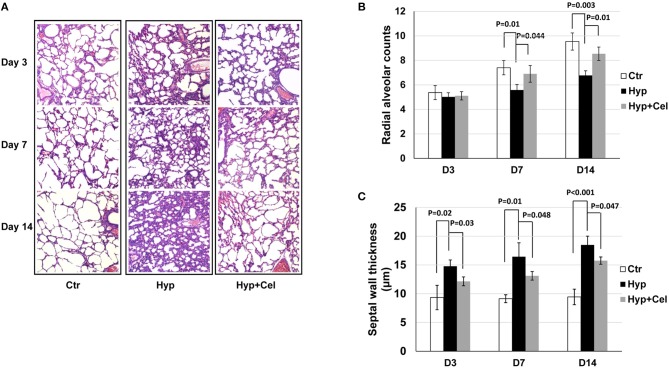
Celecoxib attenuated hyperoxia-induced impaired alveolarization in neonatal SD rats. **(A)** H&E staining of lung tissue sample sections of neonatal SD rats. **(B)** The radial alveolar counts (RACs) of rats exposed to hyperoxia reduced significantly on day 7 and day 14, which was increased significantly with celecoxib treatment. **(C)** Alveolar septal thickness (ST) of rats exposed to hyperoxia increased significantly, which were reduced significantly with celecoxib treatment.

The histologic analyses were assessed in the lungs of neonatal rats exposed to normoxia (21% O_2_), as well as in the lungs of rat exposed to hyperoxia (85% O_2_), where a pronounced arrest of alveolarization has been noted (20, 22). As shown in Figure 1A, lung development was disrupted by hyperoxia in neonatal rats compared with control group. However, the damage of the lungs by hyperoxia was alleviated with the treatment of celecoxib. To better define the effect of hyperoxia and celecoxib on lung development, RAC and ST were measured. In the control group, RAC increased gradually. However, in pups exposed with hyperoxia, the RAC was significantly lower on day 7 and day 14 (Figure 1B). Moreover, hyperoxia exposure increased the ST (Figure 1C). In contrast, celecoxib intervention increased the number of RAC, and reduced ST values (Figures 1B,C). Taken together, celecoxib improved lung development and alveolarization which was disrupted by hyperoxia exposure, as denoted by decreased number of alveoli and increased septal thicknesses of the alveoli.

### Effect of Celecoxib on Hyperoxia-Induced Inflammation

**Figure 2:**
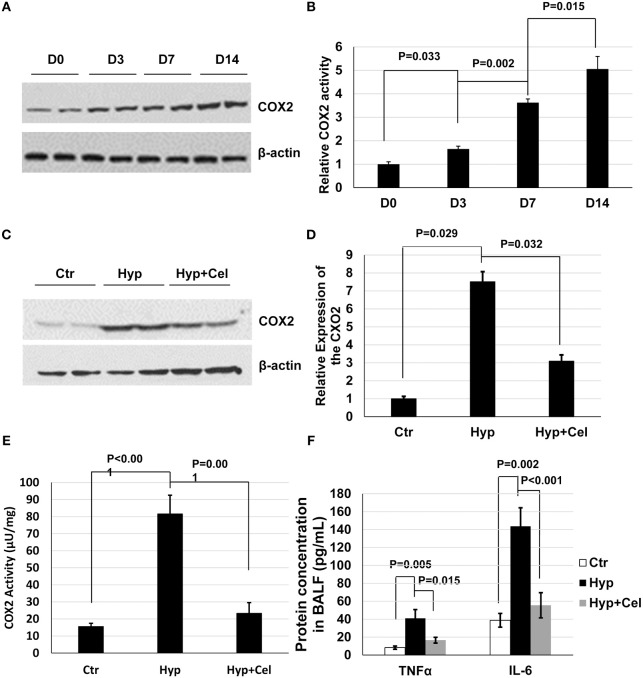
Celecoxib alleviated the hyperoxia-induced lung inflammation in neonatal SD rats. **(A)** A representative western blotting result of COX2 expression, and **(B)** COX2 activity pattern in neonatal rat lungs exposed to hyperoxia. **(C)** A representative western blotting result of COX2 expression level in lung tissue samples of three rat groups. **(D)** The densitometry of the COX2 expression western blotting results was performed using Image J and normalized to COX2 expression level in control group. **(E)** COX2 enzymatic activity was measured using COX2 activity assay kit. **(F)** The protein expression level of TNFα and IL-6 was measured in BALF in three rat groups. **(C–F)** Day 14.

Given the importance of COX2 and inflammation in BPD. We first examined COX2 expression and activity. As shown in Figures 2A,B, COX2 expression and activation was induced incrementally after sustained hyperoxia exposure in neonatal rat lungs. After celecoxib treatment, COX2 expression was modestly reduced when examined on day 14 (Figures 2C,D), and COX2 activity was dramatically induced by hyperoxia and reduced by celecoxib treatment (Figure 2E). To further evaluate the effect of celecoxib on inflammation, the level of pro-inflammatory factors was measured. As revealed in Figure 2F, hyperoxia exposure increased the secretion of TNF-α and IL-6 in BALF, and celecoxib significantly decreased the protein levels of these pro-inflammatory factors.

### Effect of Celecoxib on Apoptosis

**Figure 3:**
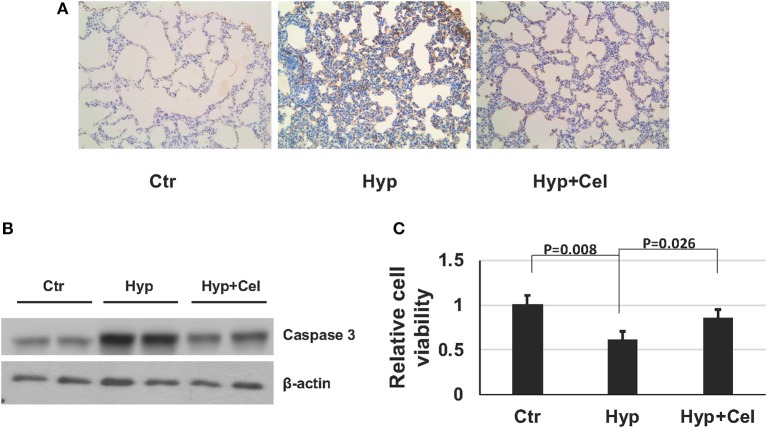
Celecoxib rescued hyperoxia induced apoptosis in lung tissue in SD rats and A549 cells. **(A)** Caspase-3 IHC staining of lung tissues of three rat groups on day 14. **(B)** Representative western blotting of activated caspase 3 expression level in lung samples of three rat groups on day 14. **(C)** Cell viability assay was assessed using Celltiter-glo and normalized to the luminescence number of control group in A549 cells.

To gain insight of the effect of celecoxib on cell growth and survival during hyperoxia-induced lung injury. We examined apoptosis of alveolar cells by IHC. As shown in Figure 3A and Table 1, there were more apoptotic alveolar cells and fewer alive cells of the tissue in a certain area in hyperoxia exposed group comparing with that in control group. Interestingly, there are more alveolar cells in total and fewer apoptotic cells in celecoxib treatment group compared with hyperoxia group. Furthermore, the western blotting result also confirmed the finding (Figure 3B). Additionally, A549 cells were cultured either in regular condition or exposed to 85% oxygen air with or without celecoxib for 72 h to confirm this finding. In accordance with the IHC results in neonatal rat lungs, there were more cell death and apoptosis in hyperoxia exposed cells comparing with control cells, and celecoxib reduced the cell death and apoptosis as shown in cell survival assay (Figure 3C).

**Table 1:** The summary of the cell numbers of the apoptosis marker stained lung tissue sections.

**Group**	**Time point**	**Number of live cells**	**Number of apoptotic cells**
Day 3	I	257.67 ± 4.04	6.67 ± 0.58
	II	187.67 ± 4.93^a^ (*P* < 0.001)	74.33 ± 4.73^a^ (*P* < 0.001)
	III	209.67 ± 5.51^b^ (*P* = 0.007)	22 ± 2.65^b^ (*P* < 0.001)
Day 7	I	256.67 ± 4.93	8.67 ± 1.53
	II	171.33 ± 7.09^a^ (*P* < 0.001)	75.67 ± 9.87^a^ (*P* < 0.001)
	III	209.67 ± 8.74^b^ (*P* = 0.004)	19.33 ± 4.16^b^ (*P* < 0.001)
Day 14	I	260 ± 7.211	9.33 ± 3.21
	II	166.33 ± 7.57^a^ (*P* < 0.001)	86.67 ± 6.8^a^ (*P* < 0.001)
	III	221.67 ± 11.37^b^ (*P* = 0.003)	18.33 ± 3.21^b^ (*P* < 0.001)

a *p-value was calculated when comparing the numbers of group II and group I at the same time point*.

b *p was calculated when comparing numbers of group III and group II at the same time point*.

### Effect of Celecoxib on NF-κB Expression and Activity

**Figure 4:**
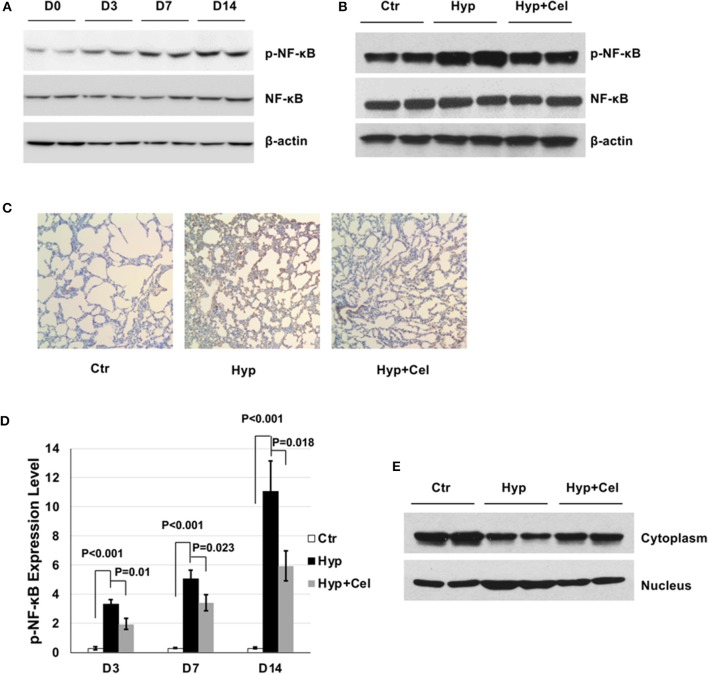
The effect of celecoxib on NF-κB activity in neonatal SD rat lungs. Representative western blotting of NF-κB phosphorylation in neonatal rat lungs **(A)** in BPD group on day 0, 3, 7, and 14 and **(B)** in the three rat groups on day 14. **(C)** p-NF-κB IHC staining of lung tissues of three rat groups on day 14. **(D)** Scoring of the p-NF-κB IHC staining slides of three rat groups on day 3, 7, and 14. **(E)** Representative western blotting of NF-κB expression in cytoplasm and nucleus of lung tissue of three rat groups on day 14.

To investigate the effect of celecoxib on the NF-κB pathway. First, we examined the NF-κB phosphorylation level in the rat model of BPD on day 0, 3, 7, and 14 by western blotting. As shown in Figure 4A, sustained hyperoxia exposure resulted in increasing NF-κB phosphorylation level in neonatal rat lungs. However, celecoxib treatment repressed NF-κB phosphorylation on day 14 (Figure 4B). To confirm this finding, we performed IHC to detect the NF-κB phosphorylation level. As shown in Figure 4C, NF-κB phosphorylation exhibited the same pattern as it was in the western blotting on day 14. Quantitation of the IHC slides on day 3, 7, and 14 further supported these findings (Figure 4D). It has been reported that enhanced NF-κB activity is either through increased nuclear translocation or transactivation (23, 24). To further investigate the inhibitory effect of celecoxib on the enhanced NF-κB phosphorylation, we compared the NF-κB levels in cytoplasm and nucleus extracts. We observed that there was increased NF-κB transported into the nucleus and less NF-κB expression in the cytoplasm with hyperoxia exposure and celecoxib had the opposite effect (Figure 4E). Taken together, these data suggest that NF-κB is activated by hyperoxia through increased nucleus translocation during the pulmonary inflammation and inhibited by celecoxib.

**Figure 5:**
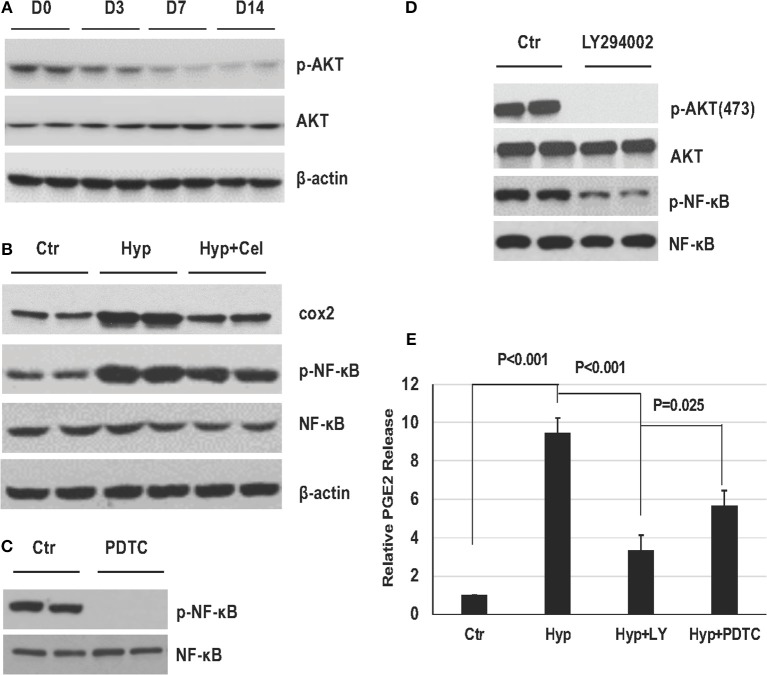
The role of NF-κB pathway in established *in vivo* and *in vitro* hyperoxia models. **(A)** AKT phosphorylation in neonatal rat lungs exposed to hyperoxia on day 0, 3, 7, and 14. **(B)** Representative western blotting of COX2 expression level, NF-κB phosphorylation levels of A549 cells in three groups. **(C,D)** NF-κB and AKT phosphorylation levels detected by western blotting with or without PDTC and LY294002 in A549 cells. **(E)** PGE2 release levels in A549 cells before and after hyperoxia exposure with or without PDTC and LY294002.

PI3K/AKT pathway has been reported to be of the upstream molecules activating NF-κB. Therefore, we were interested to access AKT phosphorylation status in the rat model during BPD development. As shown in Figure 5A, AKT phosphorylation was modestly decreasing in the rat lungs with prolonged exposure of hyperoxia. Further, to elucidate the role of NF-κB pathway in the hyperoxia-induced lung injury, A549 cells were cultured either in regular condition, or in hyperoxia condition with or without celecoxib. Figure 5B showed COX2 expression and NF-κB phosphorylation levels were induced under hyperoxia condition and celecoxib repressed their induction. Then, A549 cells were treated with either NF-κB inhibitor (PDTC) or PI3K/AKT inhibitor (LY294002) for 1 h before hyperoxia exposure. Pretreatment of the cells with PDTC and LY294002 dramatically inhibited their phosphorylation, respectively (Figures 5C,D), and the increased PGE2 release was greatly reduced by the two inhibitors treatment (Figure 5E). These further indicate that NF-κB pathway plays a principal role in the development of BPD.

### Effect of Celecoxib on AQP1 During Hyperoxia-Induced Lung Injury

**Figure 6:**
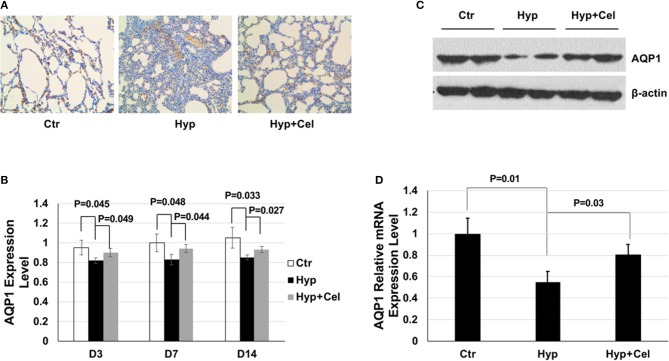
The effect of celecoxib and hyperoxia on AQP1. **(A)** AQP1 IHC staining of lung tissues of three rat groups on day 14. **(B)** Scoring of the AQP1 IHC staining slides of three rat groups on day 3, 7, and 14. **(C)** Representative western blotting of expression levels of AQP1 in lung tissue samples of three rat groups on day 14. **(D)** AQP1 mRNA expression level detected by qPCR using lung tissue from three rat groups and normalized to the mRNA expression level of the control group on day 14.

AQP1 is important in maintaining pulmonary fluid transportation and apoptosis process (14, 15). Therefore, we studied the effect of celecoxib on AQP1. IHC results showed that AQP1 protein expression level decreased in newborn rat lungs exposed to hyperoxia and increased after celecoxib treatment on day 14 (Figure 6A). Quantitation of the IHC slides on day 3, 7, and 14 demonstrated that AQP1 expression level increased gradually over time and hyperoxia exposure suppressed the expression of AQP1, whereas celecoxib treatment reversed the inhibition effect of AQP1 by hyperoxia (Figure 6B). Western blotting was also conducted to examine AQP1 expression. The AQP1 expression trend was the same to that shown in the IHC results (Figure 6C). To further study whether the changes of AQP1 was regulated at transcriptional or translational level, qPCR was conducted. As shown in Figure 6D, *AQP1* mRNA expression also decreased after hyperoxia exposure and increased after celecoxib treatment.

## Discussion

With the development of the Newborn Intensive Care Unit (NICU), neonatal care has improved rapidly. Mechanical ventilation plays a crucial role in saving newborn lives. However, as more and more children are saved, the corresponding lung injury problem has raised attention (25). Chronic pulmonary inflammation and pulmonary interstitial fibrosis caused by long-term hyperoxia exposure are key factors in the development of BPD in neonates, especially preterm infants (3, 26, 27). Even with the development of new therapy, BPD is still incurable and unpreventable (4). In this study, we sought to delineate the mechanism of protective effect of celecoxib and provide a theoretical basis for prevention and treatment of BPD.

In our study, disrupted lung development along with changes of RAC and ST were observed in newborn SD rats exposed to hyperoxia. Celecoxib treatment rescued these pathological changes, which indicates that COX2 plays an essential role in hyperoxia-induced lung injury. The previous study has reported that COX2 expression was induced in preterm infants who developed BPD and mice BPD model (5, 28). We further investigated the induction of COX2 by hyperoxia in detail and demonstrated that COX2 expression and activation were induced incrementally by prolonged hyperoxia exposure. COX2 expression was shown to be associated with the expression of inflammation marker, BALF protein level and chemoattractant, which suggest the pivotal role of COX2 in inflammation during lung injury (5). Accordingly, we noted that hyperoxia increased the expression of TNF-α and IL-6 which are important inflammatory mediators during the development of BPD. Additionally, inhibition of COX2 reduced the expression level of TNF-α and IL-6, which provides an opportunity for use COX2 as potential target of treating and preventing BPD.

Apoptosis is one of the important characteristics in lung injury caused by hyperoxia exposure (29). Apoptosis results in alveolar structure destruction, recruitment of inflammatory cells, and enhanced vascular permeability, which leads to the development of BPD (30). Inflammatory cytokine TNF-α can induce apoptosis during inflammation and high level of TNF-α leads to the development to BPD (3). Accordingly, we observed that apoptosis of alveolar cells was significantly higher in the hyperoxia group than that in the control group, and celecoxib significantly reduced the number of apoptotic cells. It suggests that celecoxib alleviates hyperoxia-induced lung injury through rescuing the alveolar cells from apoptosis and reduced the change of the development into BPD.

NF-κB signaling pathway regulates the expression of a large array of genes involved in inflammation and angiogenesis (31). Previous studies have demonstrated infiltrated inflammatory cells (macrophages, lymphocytes, etc.) and inflammatory factors (TNFα, IL-1β, IL-6, IL-8, etc.) were induced during the process of lung injuries caused by hyperoxia, which can activate NF-κB expression (31–35). And the activation of NF-κB was increased in experimental models exposed to hyperoxia, which indicates the key role of NF-κB in lung disease and pulmonary inflammation (31, 34, 35). In this study, we found that COX2 inhibition affected NF-κB signaling pathway in the inflammatory response in our BPD model. We observed that NF-κB phosphorylation was enhanced by hyperoxia, and repressed by celecoxib through regulating NF-κB nucleus translocation. In sharp contrast to our finding, Brian Poligone and Albert Baldwin have shown that COX2 blocked NF-κB nuclear translocation in HT-29 cells upon TNFα treatment (36). The precise reason for the discrepancy is unclear at the moment. However, it is human epithelial cells that were used in their study and TNFα was the only stimuli, and we used SD rats which has a more complex system to respond to hyperoxia and there were more inflammatory factors involved. Additionally, we utilized the A549 cells exposed to hyperoxia as our cell line model and observed that the induction of COX2 expression and NF-κB phosphorylation, which was inhibited by celecoxib treatment. If NF-κB has a causal role in the development of lung injury provoked by hyperoxia, one would expect to see blocking NF-κB activation rescues the disturbance of the lung injury. Indeed, we noted that NF-κB inhibitors repressed the PGE2 release induced by hyperoxia. PI3K/AKT pathway has been reported to be involved in the EP2 pathway and to be upstream of NF-κB phosphorylation (34, 37). Our *in vitro* A549 cell model also confirmed that PI3K/AKT can positive regulate NF-κB phosphorylation. Interestingly, however, decreasing AKT phosphorylation was detected in the rat lungs in the development of BPD, which seemed that the *in vivo* AKT results seemed discordant with *in vitro* finding. There were the following reasons: first, although the decreased AKT resulting in decreased NF-κB, there were other signaling pathways may be involved in the development of hyperoxia-induced lung injury, such as MAPK pathway, which can be activated by hyperoxia and upregulate the phosphorylation of NF-κB (38–42). So, the inhibitory effect on NF-κB caused by decreased AKT may be overcome by other pathways during the development of BPD. Second, Ahmad et al once have reported that AKT phosphorylation was induced by hyperoxia up to 48 h, and then started to decrease after 48 h in primary human lung microvascular endothelial cells (43). This finding was also consistent with ours, we found that decreased AKT phosphorylation was detected on day 3, 7, and 14. Third, AKT activation has been reported to be negative regulator of apoptosis (44), the effect of decreased AKT were also aligned with the effect that hyperoxia induced lung apoptosis in our BPD model. These results suggest that there are many signaling pathways involved in the development of hyperoxia induced neonatal hyperoxic lung injury and provide further evidence that NF-κB pathway contributes to the development of BPD and serves as a potential therapeutic target for treating inflammation and BPD.

AQP1 mainly transports fluid in interstitial lung and is closely related to pulmonary edema (15, 16). AQP1 expression was also shown to be repressed in lung tissue of mice treated with LPS which induced lung injury (45, 46). Furthermore, a high level of AQP1 expression has been reported in many carcinomas, and AQP1 overexpressing cells showed resistance to apoptosis, which suggests the anti-apoptotic role of AQP1 (18). Consistent with this, our results revealed that compared with the air control group, the expression of AQP-1 in hyperoxia group was significantly decreased, and the expression level of caspase 3 was increased in this group, meanwhile, compared with hyperoxia group, the expression of AQP-1 in celecoxib treatment group was significantly increased, while the activated caspase 3 in this group was decreased, which suggests that AQP1 may have preventative effect on apoptosis and degree of pulmonary injury during BPD development.

In summary, our data demonstrated that hyperoxia induced apoptosis and inflammation in rat BPD model. We observed that increased COX2 expression and activity, increased NF-κB phosphorylation and nucleus translocation, reduced AQP1 expression are the underlying mechanism for the onset of apoptosis and inflammation during hyperoxia-induced lung injury. Fortunately, celecoxib treatment alleviated all these pathological changes by inhibiting COX2, NF-κB and AQP1 pathway, which indicated that celecoxib plays a seminal role in attenuating neonatal hyperoxic lung injuries, such as impaired alveolarization, increased cell apoptosis, and inflammatory responses. The application of our findings is that celecoxib may be a promising pharmacological agent against BPD. Considering the possible toxicity of celecoxib and the fact that there is no report for its use in BPD, further studies are necessary to evaluate the safety and efficacy of celecoxib in the treatment of BPD. Such studies may broaden the therapeutic range of BPD against which celecoxib is shown to have therapeutic potential, and improve the safety of celecoxib treatment for human newborn clinical use.

## Ethics Statement

The animal experiments were carried out according to the guidelines established by the Animal Ethics Committee of the Affiliated Hospital of QingDao University.

## Author Contributions

DL designed the experiments and wrote the manuscript. DL, YW, LilL, HZ, LiaL, YL, HJ, XL, and RZ performed the experiments and data analysis. All authors reviewed and approved the final version of the manuscript.

### Conflict of Interest Statement

The authors declare that the research was conducted in the absence of any commercial or financial relationships that could be construed as a potential conflict of interest.
